# It's the Destination *and* the Journey—A Mapping of the Challenges in Transport and Referral for Maternal and Newborn Health in Pandemics and Beyond

**DOI:** 10.3389/fpubh.2021.612409

**Published:** 2021-04-16

**Authors:** Emma Sacks, Vanessa Brizuela, Carla Perrotta

**Affiliations:** ^1^Department of International Health, Johns Hopkins School of Public Health, Baltimore, MD, United States; ^2^Centro de Estudios de Estado y Sociedad, Buenos Aires, Argentina; ^3^School of Public Health, University College Dublin, Dublin, Ireland

**Keywords:** transport, referral, maternal and newborn health, obstetrics, COVID-19, health systems, emergencies

## Introduction

There has been an abundance of work in the last few years highlighting the importance of high-quality health systems as foundational to achieving better health outcomes (1). Quality provision of care is particularly critical for women and newborn during the perinatal and the immediate postpartum period (2). Transport issues are generally considered as important for access to care, but the quality of these transport and referral systems is equally essential. Recent systematic reviews related to the provision of intrapartum and postnatal care found that women were unlikely to accept referral if they had negative perceptions of the health facility (3) and were generally unsatisfied with delays in referral processes (4). Other studies have noted poor capacity at referring facilities (5), as well as challenges with coordination, communication, documentation, and adherence to referral protocols, especially for newborns (6).

The World Health Organization standards for quality of maternal and newborns care and for care of small and sick newborns include that women and newborns must be assessed and treated with available resources or appropriately referred, without delay at any time. Functional referral systems are thus integral at each stage: initial transport to the facilities, referral between facilities, and transportation whenever follow-up is required (7, 8). The COVID-19 pandemic has stretched the limits of health systems, including transport and referral pathways (9); the question of how women and their newborns reach these facilities becomes ever more urgent in this current pandemic.

## What is Referral Without a System? Just a Recommendation

Every health facility cannot not be equipped to deal with maternal and neonatal emergencies. Many countries have established regionalization of obstetric care, focusing on a few tertiary hospitals able to care for obstetric and perinatal emergencies, using a network of primary and secondary facilities to assess, stabilize, and refer (10). Referral systems call on inter-sector approaches that involve transportation, communication, and finance sectors and a coordinated response from the referring facilities and the receiving ones (11). This requires on going national or regional assessments of population needs and health system capabilities and coordinated communication.

Seamless communication requires adequate staffing at the referring and receiving facilities, with clear roles before, during, and after transport (7, 8). Within both referring and receiving facilities, it may not be clear whose responsibility is it to assist or coordinate the process, and choices may be made *ad hoc* rather than through a centralized system. In a study surveying decision-makers in Argentina, 75% stated that issues with coordination between facilities was a key barrier to timely referrals (12). A study in the Netherlands identified a desire for providers at the referring facilities to be more familiar with the services and competencies at the receiving facilities (6). In both high- and low-income settings, poor coordination between facilities leads to lost time, unnecessary visits to multiple sites, and additional costs. Receiving facilities must be notified of incoming patients in order to prepare equipment, medication, and staff. In low-resource settings, reliable technological systems, such as functional landlines or sufficient mobile airtime, may be barriers to such care, but high-resource settings often have inequitable distribution of facilities and resources such that some receiving facilities are overwhelmed and others are inaccessible due to location or cost (13). A maternal and newborns mortality reduction project in Zambia and Uganda selected ambulance coordination as a key intervention to improve health outcomes (14), aiming to reduce the burden on individuals to implement and improvise. A study in a large Canadian city found unnecessary delays in neonatal transport related to a lack of immediate vehicle, blood, or bed availability. Further unpublished reports have noted a lack of precision on the timing of incoming critically ill patients to tertiary facilities, and in countries without universal health coverage such as the United States, there are many documented challenges related to insurance policies, from the necessity of traveling further to reach facilities within an insurance network to unexpected costs from emergency ambulance transport by road and by air (15).

Strengthening referral systems is not inexpensive (11, 16, 17). Beyond the purchasing or leasing of vehicles and petrol and maintaining a functional fleet, the training, certification, and supervision of drivers are also necessary for safe and stable transportation (13). Appropriate transport goes beyond sufficient number of vehicles and drivers. Oftentimes, ambulances are not fully equipped with proper stabilization medications or oxygen and incubators. A study at one tertiary facility in Nigeria found that <10% of critically-ill neonates had been transported in vehicles with oxygen or resuscitation equipment (13). Throughout the world, many patients travel to facilities in private vehicles; even in ambulances, medical providers do not always travel with the patient during referral. Other studies have found that many women and newborns are not stabilized prior to transport; many are discharged or referred with no follow-up plan (16). A mixed-methods study related to surgical capacity in Uganda reported on the common occurrence of women in obstructed labor receiving medication, but not being directed or escorted to a particular facility and left to coordinate their own transport (18). A report from Mexico documented cases of critically ill newborns transported without medical personnel present (19). Even in places where transport to a facility is free for patients, it is rare that transport is provided after discharge, for either returning home or for follow-up visits. The cost of returning to the facility has been found as a barrier to care in both high- and low-income settings (4, 20).

### Multiple Delays

The traditional framework of the three-delay model (delay in deciding to seek care, delay in reaching a health facility, and delay getting appropriate treatment) can also be applied to challenges specific to transport (21). We carried out a mapping exercise of the necessary aspects of a functional transport and referral system across each type of delay (Table 1). To reduce the first delay, there must be recognition of symptoms and knowledge of how to initiate rapid and safe transport. To reduce the second delay, vehicles must be dispatched and ambulances or other systems of transport must be deployed. The local geography, epidemiology, and political conditions must be understood in order to select appropriate methods of rapid and safe transport. In referring between facilities, coordination should be facilitated, understandable, and seamless. Equipment and staff for stabilization, triage, and treatment must be available at the referring and receiving facilities. To reduce the third delay, the facility level must meet the minimum standards to assess, treat, or refer the patient. Where post-discharge follow-up is needed, plans must be in place to assist families in returning for care. At every stage, families should be treated with respect, with clear communication and proper consent procedures, and as much involvement as possible.

**Table 1:** Facets and functions of the transport and referral system in maternal and newborn care (potentially exacerbated by the COVID-19 pandemic).

**Function**	**Requirements**	**Exacerbation due to COVID-19**
Recognition of symptoms	• Knowledgeable woman or family member, or health worker visit • Antenatal assessment with appropriate skill and technology	• Health workers may be unable to visit houses because of travel restrictions and infection-prevention measures • Women may have had fewer antenatal care visits and be less prepared
Initiate transport	• Knowledge or whom to contact for information and care • Phone number or SMS to call for help • Free hotline or airtime	• Fear of travel and infection • Unclear regulations about the definition of “essential” or “exempted” health care • Unclear knowledge of what constituted an “essential” health intervention • Informal payments by staff may not be available during times of economic hardship of staff
Dispatch center	• Proficiency in multiple languages • Ability to send ambulance or health care worker to home or referring health center	• Fewer staff available • Center overwhelmed with COVID-19 cases
Ambulances	• Vehicle must be maintained and available • All costs must be covered (including fuel) • Woman/newborn must be stabilized • Ambulances must be sanitized frequently • Boats, helicopters, or other modes of transport may be required where there are no passable roads	• Fewer drivers available • Drivers must be protected from COVID-19 exposure (space and personal protective equipment, PPE) • Restrictions on travel because of lockdowns • Ambulances may be repurposed for COVID-19 patient transport • Ambulances need more decontamination • Increased risk of COVID-19 exposure due to ineffective infection control • Oxygen may be less available because of need for COVID-19 patients • Staff may be redeployed for COVID-19 patients • Families may not be allowed to accompany woman/newborn
Basic level facilities	• Rapid assessment and management • Equitable triage based on severity • Providers and medications must be available • Ability for further transport/refer to other facilities • Ability to stabilize pre-transport • Must coordinate referral with dispatcher and receiving facility • Detailed record keeping	• Fewer staff available • Delays may occur if entry only permitted after COVID-19 screening • Triage more complex with COVID-19 patients • Oxygen and ventilators may be prioritized for COVID-19 • More crowding and fewer beds available • Food may not be served due to COVID-19 concerns • Increased risk of COVID-19 exposure if no running water or ineffective infection control • Potential shortages of medications due to supply chain disruption
Emergency-level facilities	• Effective handover to receiving facility • Equitable triage based on severity • Assessment and management • Detailed record keeping • Registration for inpatient admittance • Sufficient space, technology, and skill for critical care • Private, hygienic spaces for postoperative recovery • Proper consent for procedures • Waiting room availability for families or support persons • Civil registration information for newborns (birth and death certificates)	• Fewer clinical and administrative staff available • Delays may occur if entry only permitted after COVID-19 screening • Triage more complex with COVID-19 patients • Oxygen and ventilators may be prioritized for COVID-19 • More crowding and fewer beds available • Food may not be served due to COVID-19 concerns • Increased risk of COVID-19 exposure if no running water or ineffective infection control • Potential shortages of medications due to supply chain disruption • Reliance on technology is inequitable in poor facilities
Neonatal intensive care	• Competent staff • Appropriate pediatric equipment • Facilitation of breast milk/human milk feeding • Facilitation of skin-to-skin care when possible • Family access to newborn • Rooming-in whenever possible	• Fewer staff available • Equipment may be delayed due to supply chain issues • Limited oxygen availability because of COVID-19 prioritization • Newborns may be kept separate from parents due to COVID-19 concerns • Limited access of family members to neonatal intensive care unit (NICU) • Space may have been repurposed for COVID-19 patients
Post discharge follow-up	• Information about how/who to follow-up, by phone or in person • Knowledge about danger signs • Transport should be provided free	• Fewer staff available • Limited ability for in-person follow-up • Families may be discouraged from seeking postnatal care • Fear of exposure of newborn to pathogens
Respectful family communication and involvement	• Health facility must be a welcoming and supportive environment • Health workers should be trained, supported, and respected themselves • Families must be given transparent information and involved in decision-making • Translators and interpreters should be available • All women should be respected regardless of socioeconomic status, ethnicity or race or origin, age, preexisting conditions or disability, etc.	• Family members may not be able to be present • Communication technology may not be equitably distributed • Fewer translators available • PPE hinders facial expressions, eye contact, lip reading • Few social workers available • Health care workers are overstressed • Guidelines may be changing rapidly • COVID-19 patients may be stigmatized

In most countries, obstetric and newborns transport is still largely the responsibility of the woman and her family. Women may already face limited decision-making power or control of funds for care-seeking and transport, and this may be exacerbated further during acute medical emergencies, when there are unexpected additional costs or uncertain insurance coverage. Even where referral is initiated by a health facility, many women and their families are responsible for getting themselves to tertiary facilities, even when the condition is time-critical. As many as 60–80% of obstetric patients in one study in Ghana arrived in the referral hospital by taxi or taken by family or friends (22). Families coordinating their own referrals may face challenges with language and literacy, including the reasons and the level of urgency. Women and their families may not know where to go; they may not have the funds to pay for transportation or the time to arrange the safest or lowest cost options, especially if they or their newborns are in critical condition (6, 7, 13, 16).

There have been reports of health workers and hospital staff arranging and paying for referral, volunteering time to coordinate transport, or offering their existing personal phone and professional networks (23, 24). Thus, the system relies on health workers to go beyond their job duties, adding to both their burden and that of patients.

### Respectful Care and Transport

Women and newborns deserve respectful care during each step on their journey to receive health services: during travel, upon arrival, and in the case of being referred to other facilities. Safe, reliable transport is as important as other healthcare needs (25).

Even when transport is provided, there may be little or no assistance provided to facilitate the transfer and limited emotional or psychosocial support in a stressful time. In many cases, family members are unable to travel with the woman or newborns; at times, the woman or the newborns may not be stabilized before travel or accompanied by a health care professional, jeopardizing their health on the journey and leaving a vulnerable patient alone (13, 16). While rare, there have been reports of newborns separated from their parents in order to receive medical care at a different setting, thus leaving the newborns alone at a critical time for survival and limiting the parents' ability to consent for their child's care (19).

The impact of mistreatment during transport and referral for critical mothers and newborns, while unknown, is likely to be detrimental given the documented negative health effects of mistreatment during childbirth on maternal and newborns health (26). The receipt of poor quality care or poor treatment at previous health facility visits, including lack of assistance with transport, may deter care utilization, especially where the direct and indirect costs of care-seeking may be high. Women and families have been found to bypass the nearest options for perceived better quality care elsewhere, potentially leading to longer journeys, higher risk, and more delays (11). Families may completely avoid care-seeking (or delay until the health condition is more severe) due to stigma or fear of mistreatment upon arrival, as many women are blamed for arriving at facilities “late” and with complications, and some are even fined (25). Under-resourced and overcrowded facilities are more likely to treat women poorly or deny care, including turning people away, neglecting them once they arrive, or demanding payments before providing services or discharging (27, 28). Avoidance or denial of care may be affecting the most vulnerable, further increasing inequity in access to safe and respectful care.

Finally, the lack of a transport system to morgues or funeral sites leaves some families without a respectful option after the death of a family member (including stillbirths and newborns) at a facility or in transit (29).

## The Double Burden During Population Emergencies and Crises

This already challenging situation of maternal and neonatal transport is exacerbated by population emergencies, such as the one posed by the COVID-19 pandemic.

The COVID-19 pandemic has impacted health systems in low- and high-resourced settings alike, including the different aspects of transport and referral systems. As part of our mapping exercise, we delineated the various facets of a transport and referral system and how the COVID-19 pandemic is exacerbating or may exacerbate each step along the transport and referral pathway (Table 1).

Successful referral systems for obstetric and perinatal emergencies during population emergencies and crises require multiple contingencies along the pathway. Any weakness in the process can result in increased delays or failure to reach a facility, potentially leading to excess deaths. Existing barriers will only be exacerbated during emergencies, both in acute events and protracted ones. In some cases, travel has become unsafe due to fear of contagion (30) or impossible because of restrictions, which may result in fewer ambulances for obstetric and neonatal emergencies, fewer available drivers or mechanics, fewer options for public transport, and delays such as police roadblocks and reduced circulation for public and private transport. A recent WHO survey of health system disruption resulting from COVID-19 indicated that a wide range of health services, including those for obstetric and neonatal emergencies, have been negatively impacted by the pandemic (9).

Dedicated transport vehicles are often needed during infectious disease outbreaks (31, 32), but in weak health systems, this will come at the expense of primary care and essential services. Medical transport of women and newborns needs to be prioritized in population emergencies, and to the greatest extent possible, provided for free and with ensured safe passage (33). The importance of well-functioning dispatch services with active collaboration between referral levels and across sectors, formalized communication strategies, and transport arrangements becomes paramount. While the unique epidemiologic situation in each setting will determine the specific policies, agreed-upon, adaptable protocols for referring and receiving facilities that respond to global standards for quality care are needed. This includes supervision and accountability for performance and additional support during crises.

## Building Better Transport Systems

A stronger transport system will result from an integrated and inter-sectoral approach. To strengthen systems, governments and health programs need to monitor the process by performing audits, collecting utilization and performance data, and being responsive to the needs of women and their families, as well as health workers and other support staff. More work is needed to continue improving and measuring effective referral and transportation interventions, policies, and strategies (4, 11). The COVID-19 pandemic has shed light on the existing weaknesses in transport and referral systems and calls on the global maternal and perinatal health community to advocate and ensure that transport methods are safe, available, and effective.

## Author Contributions

This article was first conceived by VB and ES. ES wrote the first draft of the manuscript. All authors contributed equally to the final write-up. All authors approved the final version of the manuscript.

## Conflict of Interest

The authors declare that the research was conducted in the absence of any commercial or financial relationships that could be construed as a potential conflict of interest.
